# Supplementary material for: Neural response generation for task completion using conversational knowledge graph
Source: PLoS One. 2023 Feb 9;18(2):e0269856. doi: 10.1371/journal.pone.0269856 (PMC9910720; doi:10.1371/journal.pone.0269856)
Supplement: Attachment — Submitted filename: Response_revision_1.pdf [file pone.0269856.s001.pdf]

# Detailed response to reviewers

We thank the Editor, Associate Editor and Reviewers for their insightful suggestions and feedbacks. This paper has now been revised based on the comments received. In the revised version, we have tried to address all the concerns in the best possible ways. The comments and/or feedback are all very helpful, and we are appreciative of such constructive suggestions. We respond to the comments of the reviewers point-wise. We also highlight the changes in blue text in the manuscript. We hope the revisions would improve the quality of our paper.

The major changes incorporated in the current paper are enumerated as follows:

- We have expanded the abstract as suggested
- We have expanded the introduction with clear contributions of our work along with background and scope
- We have expanded the literature review and added the papers suggested by the reviewers
- We have expanded the methodology section by adding model architecture, workflow and flowchart
- We have added another automatic evaluation metric to evaluate our system
- Added detailed discussion in the analysis section along with the limitations

## Response to the Comments of Reviewer 1

- **Query 1:** There are several data used in this paper, but not provided the suitable citations for them. Recommended to provide the suitable citations
  **Response 1:** We appreciate the suggestion by the reviewer. We have used only one dataset consisting of several domains in our research. This paper proposing this dataset is cited in Section 4.1.

- **Query 2:** . Provide the Bi-LSTM Encoder Decoder with illustrative example for better understanding the model
  **Response 2:** As suggested by the reviewer we have provided the illustrative example for the model in form of Figure 1,2,3 and 4 for this purpose. The detailed description is also provided in the 3.2 to 3.8.

- **Query 3:** . Discuss the time complexity for the Algorithm 1 discussed in this paper
  **Response 3:** As suggested by the reviewer we have provided this discussion in Section 3.9.

- **Query 4:** The discussion under Section 3.6 to 3.8 are very limited. Recommended to provide the details discussion and comparative analysis.
  **Response 4:** As advised we have provided the details and comparative analysis in the corresponding sections.

- **Query 5:** . The results of the paper are very limited. Recommended to provide the different metrics for each dataset. It is recommended to provide the suitable reasons for the superior performance of the proposed models.
  **Response 5:** We have added another automatic evaluation metric to measure our results. Unlike the previous metrics where the word-overlap is measured, this metric measures the semantic similarity between the desired and produced output. We have provided the detailed explanation and analysis for the same in Sections 5.1 and 5.2.

- **Query 6:** . Discuss the limitations of the proposed model and possible future extensions on it.
  **Response 6:** As suggested we have added these discussions in Section 5.2 and 6.

- **Query 7:** .Summarize the related work using a table with the benefits and limitations of the existing works. It is also recommended to discuss which among the existing limitations are addressed in this paper.
  **Response 7:** As suggested, we have added Table 1 for this purpose.

## Response to the Comments of Reviewer 2

- **Query 1:** Abstract- Highlight the novelty aspect after Aim/Objective of the paper. Add in the last lines in what %age and in what parameters the proposed methodology is better and as compared to existing techniques and what is the overall analysis of the proposed technique.
  **Response 1:** As suggested, we have expanded our abstract to reflect the points.

- **Query 2:** . Introduction needs to be more broad with regard to the Background, SCOPE, Problem Definition and even other related highlights. Add Objectives of the paper in Points. Add Organization of the paper.
  **Response 2:** We have added the suggested details in the introduction section (Section 1).

- **Query 3:** Related works needs to be more and min 15-25 papers should be there in Related works. ADD in the last lines what overall technical gaps are observed that led to the design of the proposed methodology.
  **Response 3:** As suggested we have expanded the related works. We have also added Table 1 highlighting the benefits and limitations of previous works in comparison to our work.

- **Query 4:** Add the flowchart of the proposed methodology. Add System Model and Step by Step operation of working of proposed technique.
  **Response 4:** As advised we have added 1,2,3,4 and 6 for this purpose. The working of each of the modules are discussed in Section 3.

- **Query 5:** Add more information towards results. And perform Performance analysis with some existing techniques.
  **Response 5:** We have added another automatic evaluation metric to measure our

results. Unlike the previous metrics where the word-overlap is measured, this metric measures the semantic similarity between the desired and produced output. We have provided the detailed explanation and analysis for the same in Sections 5.1 and 5.2. The techniques against we compare i.e Bi-LSTM encoder-decoder and HRED are pre-existing techniques used for response generation.

- **Query 6:** Add Analysis based information to the paper
  **Response 6:** We have added detailed analysis in section 5.2 of the paper.

- **Query 7:** Add case study based discussion to the paper.
  **Response 7:** We have added this discussion in Section 5.2 of the paper.

- **Query 8:** Add future scope to the paper.

  **Response 8:** We have discussed the limitations and future scopes in Section 5.2 and 6.
