# Supplementary material for: Neural response generation for task completion using conversational knowledge graph
Source: PLoS One. 2023 Feb 9;18(2):e0269856. doi: 10.1371/journal.pone.0269856 (PMC9910720; doi:10.1371/journal.pone.0269856)
Supplement: Attachment — Submitted filename: response.pdf [file pone.0269856.s002.pdf]

Detailed response to reviewers

We thank the Editor, Associate Editor and Reviewers for their insightful suggestions and feedbacks. This paper has now been revised based on the comments received. In the revised version, we have tried to address all the concerns in the best possible ways. The comments and/or feedback are all very helpful, and we are appreciative of such constructive suggestions. We respond to the comments of the reviewers point-wise. We also highlight the changes in blue text in the manuscript. We hope the revisions would improve the quality of our paper.

The major changes incorporated in the current paper are enumerated as follows:

- We have expanded on the discussion regrading time complexity of the algorithms proposed in this paper
- We have created discussed the limitations and future scope of the work in a separate subsection section
- We have discussed the reasons for superior performance of our method in a new discussions subsection

Response to the Comments of Reviewer 1

- **Query 1:** The complexity analysis of the proposed work is missing in the paper.
  Response 1: We have expanded our complexity analysis in Section 3.9 under the heading Time Complexity Analysis (lines 283-398).

- **Query 2:** The future scope and limitations are not included in the paper.
  Response 2: We have added Section 5.4 named Limitations and Future Scope (lines 425-436) for this discussion.

- **Query 3:** The reasons for achieving the superior complexity of the proposed work is missing in the discussion of results.
  Response 3: We have added discussion on these points in Section 5.3 (lines 413-424)
